# Understanding of Prognosis and Estimation of Mortality in Ambulatory Patients With Heart Failure

**DOI:** 10.1001/jamanetworkopen.2026.0328

**Published:** 2026-03-03

**Authors:** Thomas M. Cascino, Grace Herron, Blair Richards, James W. Stewart, Wayne C. Levy, Geoffrey D. Barnes, Colleen K. McIlvennan, Wendy C. Taddei-Peters, Neal Jeffries, Douglas L. Mann, Josef Stehlik, Garrick C. Stewart, Keith D. Aaronson, Supriya Shore

**Affiliations:** 1 Division of Cardiovascular Medicine, University of Michigan, Ann Arbor; 2 University of Michigan Medical School, University of Michigan, Ann Arbor; 3 Michigan Institute for Clinical and Health Research, University of Michigan, Ann Arbor; 4 Department of Cardiac Surgery, University of Michigan, Ann Arbor; 5 Division of Cardiology, University of Washington, Seattle; 6 Division of Cardiology, University of Colorado, Aurora; 7 National Heart, Lung, and Blood Institute, National Institutes of Health, Bethesda, Maryland; 8 Cardiovascular Division, Washington University, St Louis, Missouri; 9 Division of Cardiovascular Medicine, University of Utah, Salt Lake City; 10 Division of Cardiovascular Medicine, Brigham and Women’s Hospital, Boston, Massachusetts

## Abstract

**Importance:**

Accurate patient understanding of prognosis is essential for informed decision-making to pursue therapies for advanced heart failure (HF).

**Objectives:**

To evaluate (1) patient characteristics associated with overestimating survival with HF and (2) whether overestimation is associated with mortality.

**Design, Setting, and Participants:**

This prospective cohort study was an exploratory secondary analysis of data from the multicenter US Registry Evaluation of Vital Information for Ventricular Assist Devices (VADs) in Ambulatory Life (REVIVAL) study. Participants were high-risk ambulatory patients with HF with reduced ejection fraction enrolled from July 2015 to June 2016. Data were analyzed from December 1, 2024, to December 16, 2025.

**Exposures:**

Patient characteristics (eg, age) and estimation index (EI), defined as the ratio of patient-estimated life expectancy to Seattle Heart Failure Model (SHFM)–estimated mean survival (EI <0.5, discordantly pessimistic; 0.5 to <1.5, concordant; and ≥1.5, discordantly optimistic).

**Main Outcomes and Measures:**

Primary outcomes were EI and 2-year all-cause mortality. Factors associated with EI were estimated using ordered logistic regression. Association between EI and mortality was assessed using a cause-specific Cox proportional hazards regression model with VAD and heart transplant as censoring events.

**Results:**

A total of 296 high-risk, ambulatory patients with chronic HF were included; 223 (75.3%) were male, and mean (SD) age was 60.1 (11.5) years. The median SHFM-estimated survival was 8.2 years (IQR, 5.1-12.1 years), and median patient-estimated life expectancy was 7.0 years (IQR, 5.0-10.0 years). In all, 98 patients (33.1%) were discordantly optimistic. Increasing EI was associated with increased mortality in the univariable model, which was attenuated with multivariable adjustment (adjusted hazard ratio [AHR] for concordant optimism, 1.21 [95% CI, 0.49-2.99] and for discordant optimism, 2.23 [95% CI, 0.94-5.33] vs discordant pessimism). Compared with discordantly pessimistic or concordantly optimistic estimates (EI <1.5), discordant optimism was associated with increased hazard of 2-year mortality (AHR, 1.98; 95% CI, 1.04-3.77) but a similar hazard for a VAD or heart transplant compared with discordant pessimism (HR, 1.24; 95% CI, 0.64-2.41) in a post hoc analysis.

**Conclusions:**

In this cohort study, discordant optimism regarding life expectancy compared with model estimates was common and associated with mortality that was not due to a lower probability of receiving a heart transplant or VAD. The findings suggest clinicians should objectively evaluate HF risk when considering advanced therapies, rather than relying primarily on patient-reported symptoms.

## Key Points

**Questions:**

How well do ambulatory patients with high-risk heart failure (HF) with reduced ejection fraction estimate their own survival compared with a validated, guideline-endorsed risk score, and is overestimation associated with mortality?

**Findings:**

In this cohort study of 296 patients with chronic HF, 33% overestimated their life expectancy by more than 50% compared with model estimates, and such overestimation was associated with higher mortality in the subsequent 2 years.

**Meaning:**

The findings suggest improving patient understanding of prognosis may enhance shared decision-making for patients with high-risk ambulatory HF.

## Teaser

This cohort study explores how well ambulatory patients with high-risk chronic heart failure estimate their survival compared with a validated risk model and whether overestimation is associated with 2-year mortality.

## Introduction

Heart failure (HF) impacts approximately 6.7 million people in the US and contributes to 1 in 8 deaths.^1^ Advanced therapies, including heart transplants and left ventricular assist devices (LVADs), extend and improve the quality of life in patients with advanced HF.^2,3^ However, these therapies are invasive, are associated with complications, and mandate lifestyle changes.^4,5,6,7,8^ To maximize their benefits, there is a narrow window of opportunity when patients are sick enough from HF and have no contraindications that would impact outcomes.^2,9,10^ Thus, the decision to pursue advanced therapies is complex and requires informed decision-making, in which clinicians and patients exchange information to select treatments aligned with the patient’s values and preferences.^11^

A critical component of informed decision-making is an accurate patient understanding of prognosis. The unpredictable course of HF makes prognosis communication challenging.^11^ Prior studies demonstrate that patients with HF overestimate their survival.^12^ One likely contributor is how clinicians discuss prognosis. Contemporary evidence suggests that a minority of patients with HF have a conversation about prognosis with their clinicians, despite most wanting these discussions early in the disease course.^13,14,15^ Furthermore, it is unclear to what extent overly optimistic perceptions prompt patients to defer care, thereby worsening outcomes. Thus, identifying characteristics associated with overestimating survival and how overestimation impacts mortality could enable the design of patient-centered tools for prognosis communication, thereby facilitating shared decision-making.

The objectives of this study were to evaluate (1) patient characteristics associated with overestimating survival regarding survival compared with validated model-predicted survival and (2) whether overestimating survival is associated with mortality. We used a prospective registry of ambulatory patients with advanced HF cared for by HF specialists at tertiary centers. We hypothesized that patients with HF who are overly optimistic about their survival may underestimate the need for advanced therapy and defer care, leading to increased mortality.

## Methods

### Data Source

This cohort study was an exploratory secondary analysis of data from the Registry Evaluation of Vital Information for VADs in Ambulatory Life (REVIVAL) study. REVIVAL was an observational, prospective cohort study of ambulatory patients with chronic HF with reduced ejection fraction. It was funded by the National Institutes of Health (NIH) and has been previously described.^16,17^ REVIVAL enrolled 400 participants across 21 advanced HF centers from July 2015 to June 2016 with follow-up for 2 years or until the occurrence of a study primary end point (LVAD, transplant, or death) to characterize the clinical trajectory of patients with HF with high-risk features. Since this was an observational registry, all treatment decisions were at the clinicians’ discretion. An independent observational study monitoring board oversaw the conduct of REVIVAL. Institutional review boards at the Data Coordinating Center at the University of Michigan and each participating center approved the study protocol. The current study was approved by the University of Michigan institutional review board. Written informed consent was obtained for all participants. This study was designed and reported in accordance with the Strengthening the Reporting of Observational Studies in Epidemiology (STROBE) reporting guideline. The data for REVIVAL have been uploaded to the National Heart, Lung, and Blood Institute’s Biologic Specimen and Data Repository Information Coordinating Center and are available per NIH policy.

### Participants

REVIVAL enrolled high-risk patients with HF with an expected 1-year event rate of death, urgent transplant, or durable LVAD (dLVAD) implant of 25%. Inclusion and exclusion criteria identified patients without contraindications to advanced therapies (eAppendix in Supplement 1). We excluded participants who lacked surveys at the baseline visit.

### Exposure and Outcomes

Outcomes of interest were the estimation index (EI) and 2-year all-cause mortality. We defined EI as the ratio of patient-estimated life expectancy to the Seattle Heart Failure Model (SHFM)–estimated (hereinafter, model-estimated) mean life expectancy based on prior work.^18^ Patient-estimated life expectancy was assessed at enrollment with the question, “Based on how you feel today and what you know about your HF, what is your best guess of how much longer you have to live? For the purpose of this question, please assume that a VAD or a heart transplant would not be possible for you.”^19^

Based on the distribution of EIs to ensure adequate sample size between groups and in consideration of what would be a clinically meaningful miscalculation of life expectancy, a 50% threshold was selected. Categorization of EI was defined by comparative differences rather than by absolute differences between patient-estimated and model-estimated survival, as comparative differences are less influenced by differences in time, and the comparative importance of differences between patient-estimated and model-estimated survival is greater when life expectancy is shorter. For example, a 1-year overestimate is more consequential when expected survival is 1 year than when it is 10 years. Expressing this as a comparative difference—EI of 2.0 for the former and EI of 1.1 for the latter—captures this difference. For descriptive purposes, we refer to an EI of 0.5 to less than 1.5 as “concordant,” as it reflects relative concordance between patient-estimated and model-estimated life expectancy; an EI less than 0.5 as “discordantly pessimistic,” as it reflects a shorter patient-estimated life expectancy compared with the SHFM; and an EI of 1.5 or higher as “discordantly optimistic,” representing a higher patient-estimated life expectancy compared with the SHFM.

### Covariates

Covariates were included that may explain EI, based on existing literature from other chronic diseases^20^ and associations with HF severity or access to dLVADs. Demographic variables included participant-reported age, race, sex, caregiver presence, income, insurance status, and educational level. Participants’ self-reported race was evaluated as Black, White, or other (included American Indian or Alaska Native, Asian, multiracial, other or none of the available NIH classification options, and unknown or undisclosed), due to the small sample size. Clinical variables included the Meta-Analysis Global Group in Chronic (MAGGIC) score (range, 0-57, with higher scores suggesting worse prognosis), Kansas City Cardiomyopathy Questionnaire overall score (KCCQ-OS; range, 0-100, with higher scores indicating better health-related quality of life [HRQOL]), and depression (defined by the 8-item Patient Health Questionnaire [PHQ-8] as a score ≥10 on a scale from 0 to 24, with higher scores indicating more severe symptoms).^21,22,23,24^ All covariates were assessed at baseline enrollment.

### Statistical Analysis

Baseline characteristics were compared across EI categories using the χ^2^ test for categorical variables and analysis of variance for continuous variables. The association between patient-estimated and model-estimated life expectancy was evaluated using a scatterplot and correlation coefficient. Next, a multivariable ordered logistic regression was performed to explore factors associated with discordant optimism with EI as the primary outcome. Explanatory covariates with a prespecified 2-sided *P* value less than .20 in univariable analyses were included in all multivariable analyses. Estimated probabilities were determined for interpretability. The proportional odds assumption of ordered logistic regression was confirmed.

Next, a cause-specific Cox proportional hazards regression was performed to determine associations of patient-estimated and model-estimated life expectancy with mortality, evaluating the components of the EI first (patient- and model-estimated mortality) and then the EI. Since we hypothesized that patients with discordant optimism might underestimate the need for advanced therapies and defer care, leading to increased mortality, the primary outcome was all-cause mortality, with dLVAD and heart transplant as censoring events. Next, a cause-specific Cox proportional hazards regression was performed to understand if patients’ discordant optimism regarding life expectancy was associated with the combined outcome of dLVAD or heart transplant.

After a review of the survival curves for the association between EI and mortality, the increase in mortality was much greater between the concordant (EI 0.5 to <1.5) and discordantly optimistic (EI ≥1.5) groups. Since EI categorization was arbitrary, we also conducted a post hoc exploratory analysis with EI as a binary outcome (EI <1.5 vs ≥1.5) to evaluate the association between survival overestimation and mortality.

Multiple sensitivity analyses were performed. First, the association between patient factors and EI as a continuous variable were explored using linear regression. The EI was heavily skewed and was thus transformed using a log function. In review of the natural log–transformed EI, there were 4 extreme outliers that were more than 4.5 SDs away from the mean and more than 2.5 SDs from the next lowest value. These 4 outliers were set to the next lowest value. Next, a sensitivity analysis was performed using KCCQ-OS as a categorical variable (0-49, 50-74, and 75-100), representing poor-fair, good, and excellent health status, respectively, to examine whether the previously described health status cut points were associated with mortality. Analyses were performed using SAS, version 9.4 (SAS Institute Inc), and Stata/MP, version 18 (StataCorp LLC). Data were analyzed from December 1, 2024, to December 16, 2025. A 2-sided *P* value less than .05 was considered to be statistically significant.

## Results

After exclusion of 104 participants who lacked surveys at the baseline visit, our final cohort included 296 high-risk, ambulatory patients with chronic HF enrolled from 2015 to 2016. The mean (SD) age was 60.1 (11.5) years; 73 (24.7%) were female and 223 (75.3%) were male. A total of 64 patients (21.6%) were Black, 216 (73.0%) were White, and 16 (5.4%) were other race. The median SHFM-estimated survival was 8.2 years (IQR, 5.1-12.1 years), and the median patient-estimated life expectancy was 7.0 years (IQR, 5.0-10.0 years). The median EI was 1.0 (IQR, 0.5-1.8). Table 1 presents the baseline characteristics by discordantly pessimistic (86 patients [29.1%]), concordant (112 [37.8%]), and discordantly optimistic (98 [33.1%]) survival estimates. Patients in the discordantly optimistic group had the longest patient-estimated life expectancy (mean [SD] of 18.8 [12.1] years) and the shortest SHFM-based model-estimated survival (mean [SD] of 6.6 [4.5] years) compared with the discordantly pessimistic group (3.7 [2.2] years and 11.6 [5.3] years, respectively) and concordant group (8.3 [4.7] years and 9.2 [4.6] years, respectively) (*P* < .001 for both comparisons). The mean MAGGIC risk score was higher (suggesting higher risk) among patients in the discordantly optimistic group than in the discordantly pessimistic and concordant groups. Patients in the discordantly optimistic group also had a higher mean KCCQ-OS compared with the other groups. The correlation between patient-estimated and model-estimated life expectancy is shown in the eFigure in Supplement 1. The correlation between patient-estimated and SHFM-estimated mean life expectancy was weak (*R* = 0.18; *R*^2^ = 0.03; *P* = .002).

**Table 1.** Baseline Characteristics of the Study Population Stratified by EI Into Discordantly Pessimistic, Concordant, and Optimistic Groups^a^

Characteristic	Patients^b^				*P* value
	Overall (N = 296)	Discordantly pessimistic (EI <0.5) (n = 86)	Concordant (EI 0.5 to <1.5) (n = 112)	Discordantly optimistic (EI ≥1.5) (n = 98)	
Age, y	60.1 (11.5)	59.2 (11.4)	60.7 (11.5)	60.2 (11.7)	.66
Sex					
Female	73 (24.7)	27 (31.4)	27 (24.1)	19 (19.4)	.17
Male	223 (75.3)	59 (68.6)	85 (75.9)	79 (80.6)	
Race					
Black	64 (21.6)	13 (15.1)	25 (22.3)	26 (26.5)	.47
White	216 (73.0)	68 (79.1)	81 (72.3)	67 (68.4)	
Other^c^	16 (5.4)	5 (5.8)	6 (5.4)	5 (5.1)	
Caregiver, No./total No. (%)	209/293 (71.3)	60/86 (69.8)	77/109 (70.6)	72/98 (73.5)	.84
Annual income, $					
<40 000	105 (35.5)	32 (37.2)	41 (36.6)	32 (32.7)	.51
40 000 to <80 000	55 (18.6)	17 (19.8)	24 (21.4)	14 (14.3)	
≥80 000	41 (13.9)	10 (11.6)	12 (10.7)	19 (19.4)	
Did not report	95 (32.1)	27 (31.4)	35 (31.2)	33 (33.7)	
Insurance status, No./total No. (%)					
Medicare, Tricare, or WC	95/294 (32.3)	33/85 (38.8)	35/112 (31.2)	27/97 (27.8)	.65
Medicaid or none	26/294 (8.8)	9/85 (10.6)	9/112 (8.0)	8/97 (8.2)	
Private, commercial, or other	147/294 (50.0)	35/85 (41.2)	59/112 (52.7)	53/97 (54.6)	
Both private and public	26/294 (8.8)	8/85 (9.4)	9/112 (8.0)	9/97 (9.3)	
Educational level, No./total No. (%)					
Grade school or high school	82/252 (32.5)	27/75 (36.0)	35/95 (36.8)	20/82 (24.4)	.29
Some college or technical school	79/252 (31.3)	23/75 (30.7)	28/95 (29.5)	28/82 (34.1)	
Associate’s degree	24/252 (9.5)	8/75 (10.7)	11/95 (11.6)	5/82 (6.1)	
Bachelor’s degree	39/252 (15.5)	12/75 (16.0)	10/95 (10.5)	17/82 (20.7)	
Graduate degree	28/252 (11.1)	5/75 (6.7)	11/95 (11.6)	12/82 (14.6)	
Patient-estimated life expectancy, y	10.4 (9.8)	3.7 (2.2)	8.3 (4.7)	18.8 (12.1)	<.001
SHFM life expectancy, y	9.0 (5.2)	11.6 (5.3)	9.2 (4.6)	6.6 (4.5)	<.001
INTERMACS patient profile					
4	27 (9.1)	5 (5.8)	9 (8.0)	13 (13.3)	.09
5	56 (18.9)	23 (26.7)	20 (17.9)	13 (13.3)	
6	115 (38.9)	32 (37.2)	39 (34.8)	44 (44.9)	
7	98 (33.1)	26 (30.2)	44 (39.3)	28 (28.6)	
MAGGIC risk score	25.1 (5.6)	23.5 (5.0)	24.9 (5.6)	26.6 (5.7)	<.001
KCCQ-OS	63.1 (20.7)	54.4 (20.5)	65.0 (19.4)	68.5 (20.0)	<.001
Depression, No./total No. (%)	81/294 (27.6)	39/86 (45.3)	29/110 (26.4)	13/98 (13.3)	<.001
LVEF, %	21 (7)	21 (7)	21 (7)	21 (6)	.93
Medical therapy					
β-Blocker	279 (94.3)	80 (93.0)	109 (97.3)	90 (91.8)	.20
ACEI, ARB, or ARNI	253 (85.5)	76 (88.4)	100 (89.3)	77 (78.6)	.06
Aldosterone antagonist	219 (74.0)	70 (81.4)	80 (71.4)	69 (70.4)	.18
Loop diuretic	270 (91.2)	72 (83.7)	103 (92.0)	95 (96.9)	.006
Vital signs					
Heart rate, BPM (n = 294)	75 (12)	74 (12)	75 (13)	76 (13)	.41
Blood pressure, mm Hg					
Systolic	107 (15)	110 (16)	107 (13)	105 (17)	.06
Diastolic (n = 295)	66 (10)	68 (10)	67 (9)	64 (11)	.04
Laboratory values					
Sodium, mEq/L	138 (3)	139 (3)	138 (3)	138 (4)	.07
Creatinine, mg/dL	1.4 (0.5)	1.2 (0.3)	1.4 (0.5)	1.5 (0.5)	<.001
Total bilirubin, mg/dL (n = 291)	0.8 (0.5)	0.7 (0.5)	0.9 (0.4)	0.8 (0.5)	.13
Outcomes					
Follow-up duration, median (IQR), d^d^	717 (300-749)	727 (410-753)	721 (403-749)	687 (238-742)	.11
Died	41 (13.8)	8 (9.3)	13 (11.6)	20 (20.4)	.06
LVAD	43 (14.5)	12 (13.9)	18 (16.1)	13 (13.3)	.36
Cardiac transplant	17 (5.7)	7 (8.1)	3 (2.7)	7 (7.1)	.20

Abbreviations: ACEI, angiotensin-converting enzyme inhibitor; ARB, angiotensin receptor blocker; ARNI, angiotensin receptor–neprilysin inhibitor; BPM, beats per minute; EI, estimation index; INTERMACS, Interagency Registry for Mechanically Assisted Circulatory Support; KCCQ-OS, Kansas City Cardiomyopathy Questionnaire overall score; LVEF, left ventricular ejection fraction; MAGGIC, Meta-Analysis Global Group in Chronic; SHFM, Seattle Heart Failure Model; WC, workers compensation.

SI conversion factors: to convert creatinine to µmol/L, multiply by 88.4; sodium to mmol/L, multiply by 1.0; total bilirubin to µmol/L, multiply by 17.104.

^a^ EI is defined as the ratio of patient-estimated life expectancy to model-estimated life expectancy, based on the SHFM.

^b^ Data are presented as mean (SD) for continuous measures and number (percentage) of patients for categorical measures.

^c^ Included American Indian or Alaska Native (1 [0.3%]), Asian (5 [1.7%]), multiracial (5 [1.7%]), other or none of the available National Institutes of Health classification options (4 [1.4%]), and unknown or undisclosed (1 [0.3%]).

^d^ Follow-up was until participant received a durable left ventricular assist device or heart transplant.

**Figure 1.**
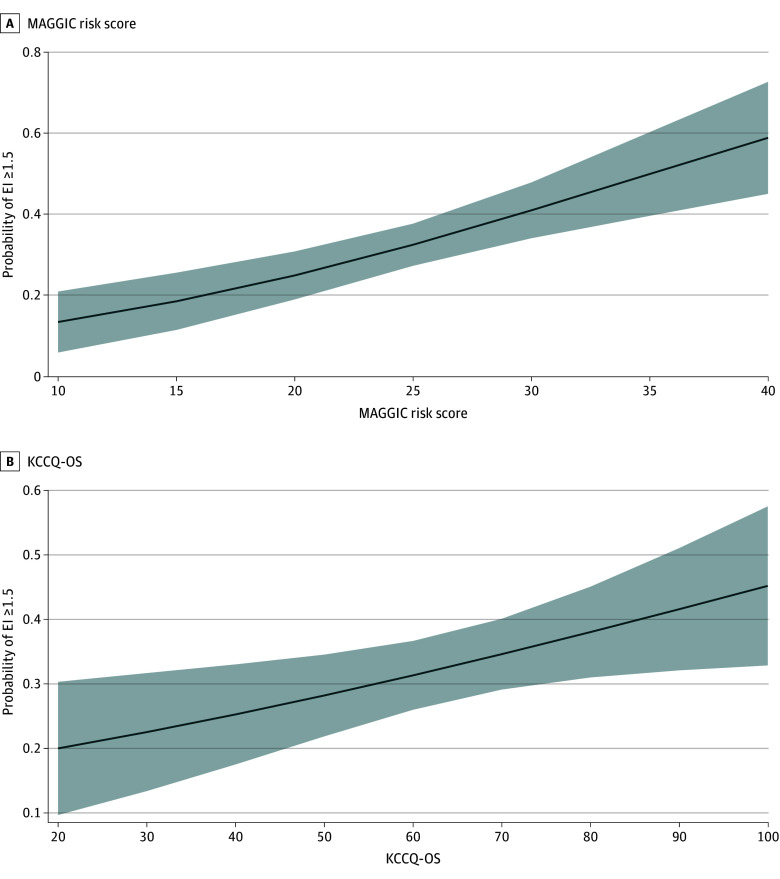
Graph Showing Adjusted Probability of Overestimated Life Expectancy by Meta-Analysis Global Group Chronic (MAGGIC) Risk Score and Kansas City Cardiomyopathy Questionnaire Overall Score (KCCQ-OS) Shading represents 95% CIs. EI indicates estimation index. Models were adjusted for sex, race, MAGGIC risk score, KCCQ-OS, and depression. MAGGIC risk score ranges from 0 to 57, with higher scores indicating worse prognosis. KCCQ-OS ranges from 0 to 100, with higher scores indicating better health status.

The results of the univariable and multivariable ordered logistic regression models examining the association between clinical and demographic characteristics and EI are shown in Table 2. In the multivariable analysis, Black race compared with White, MAGGIC risk score and KCCQ-OS per 1-unit increase, and lack of depression were associated with higher odds of having patient-estimated survival that was more optimistic than the model estimate. The estimated probabilities with 95% CIs for discordantly optimistic life expectancy by MAGGIC score and KCCQ-OS are shown in Figure 1. Patients with a higher risk for mortality per MAGGIC score and higher reported quality of life per KCCQ-OS had more discordantly optimistic estimates of life expectancy (EI ≥1.5). The estimated probability that a patient with a MAGGIC risk score of 10 (low risk) would overestimate life expectancy was 13.5% (95% CI, 6.0%-21.0%), compared with 58.9% (95% CI, 45.1%-72.3%) for a patient with a MAGGIC risk score of 40 (high risk). The estimated probability that a patient with a KCCQ-OS of 20 (higher symptom burden) would overestimate life expectancy was 20.0% (95% CI, 10.0%-30.3%) compared with 45.2% (95% CI, 32.9%-57.5%) for a person with excellent reported health status (KCCQ-OS of 100). In the sensitivity analysis using the log-transformed EI as a continuous outcome, MAGGIC score risk, KCCQ-OS, and lack of depression remained associated with EI (eTable 1 in Supplement 1).

**Table 2.** Univariable and Multivariable Associations Between Patient Characteristics and Increasing Optimism Index

Variable	Univariable		Multivariable^a^	
	OR (95% CI)	*P* value	OR (95% CI)	*P* value
Age, per year	1.01 (0.99-1.02)	.56	NA	NA
Female sex	0.63 (0.38-1.02)	.06	0.68 (0.40-1.15)	.15
Race (reference, White)				
Black	1.62 (0.97-2.71)	.07	1.79 (1.04-3.09)	.04
Other^b^	1.01 (0.40-2.58)	.98	1.72 (0.61-4.88)	.31
Caregiver (reference, none)	1.14 (0.72-1.82)	.57	NA	NA
Annual income, $ (reference, <40 000)				
40 000 to <80 000	0.88 (0.49-1.60)	.68	NA	NA
≥80 000	1.73 (0.87-3.43)	.12	NA	NA
Did not report	1.16 (0.70-1.94)	.57	NA	NA
Insurance status (reference, Medicare, Tricare, or WC)				
Medicaid or none	1.06 (0.47-2.38)	.89	NA	NA
Private, commercial, or other	1.54 (0.96-2.49)	.07	NA	NA
Both private and public	1.28 (0.57-2.86)	.56	NA	NA
Educational level (reference, grade school or high school)				
Some college or technical school	1.41 (0.80-2.50)	.23	NA	NA
Associate’s degree	0.92 (0.41-2.08)	.84	NA	NA
Bachelor’s degree	1.69 (0.82-3.49)	.16	NA	NA
Graduate degree	2.17 (0.98-4.78)	.06	NA	NA
MAGGIC risk score, per 1-unit increase	1.08 (1.04-1.12)	<.001	1.08 (1.04-1.13)	<.001
KCCQ-OS, per 1-unit increase	1.03 (1.01-1.04)	<.001	1.02 (1.00-1.03)	.02
Depression (reference, PHQ-8 score <10)^c^	0.30 (0.18-0.49)	<.001	0.46 (0.25-0.86)	.02

Abbreviations: KCCQ-OS, Kansas City Cardiomyopathy Questionnaire overall score; MAGGIC, Meta-Analysis Global Group in Chronic; NA, not applicable; OR, odds ratio; PHQ-8, 8-item Patient Health Questionnaire; WC, workers compensation.

^a^ Explanatory variables with a prespecified *P* value less than .20 in univariable analyses were included in the multivariable analysis.

^b^ Included American Indian or Alaska Native, Asian, multiracial, other or none of the available National Institutes of Health classification options, and unknown or undisclosed.

^c^ PHQ-8 score ranges from 0 to 24, with higher scores indicating more severe symptoms.

Over 2 years of follow-up, there were 41 deaths (13.9%), 17 heart transplants (5.7%), and 43 dLVADs (14.5%). Mortality increased with increasing optimism: 8 of 86 (9.3%) in the discordantly pessimistic group, 13 of 112 (11.6%) in the concordant group, and 20 of 98 (20.4%) in the discordantly optimistic group. Increasing SHFM-estimated life expectancy was associated with improved survival (hazard ratio [HR], 0.86; 95% CI, 0.79-0.93), while patient-estimated life expectancy was not (HR, 1.00; 95% CI, 0.96-1.03). The results of the univariable and multivariable cause-specific Cox proportional hazards regression models are shown in Table 3. The association between EI and mortality was not statistically significant after multivariable adjustment (adjusted HR [AHR] for concordant optimism, 1.21 [95% CI, 0.49-2.99] and for discordant optimism, 2.23 [95% CI, 0.94-5.33] vs discordant pessimism). Patients in the discordantly pessimistic group (reference) were as likely to receive advanced therapies (dLVAD or heart transplant) as patients in the discordantly optimistic group (HR, 1.24; 95% CI, 0.64-2.41) (eTable 2 in Supplement 1). Lower KCCQ-OS was associated with receiving advanced therapies (HR per 1-unit increase, 0.98; 95% CI, 0.97-0.99).

**Table 3.** Univariable and Multivariable Associations of Estimation Index With Mortality

Variable	Univariable (n = 296)		Multivariable (n = 293)^a^	
	HR (95% CI)	*P* value	AHR (95% CI)	*P* value
**Model with 3 EI levels (<0.5, 0.5 to <1.5, and ≥1.5)^b^**				
EI (reference, discordant pessimism)				
Concordant	1.26 (0.52-3.05)	.60	1.21 (0.49-2.99)	.68
Discordant optimism	2.54 (1.12-5.77)	.03	2.23 (0.94-5.33)	.07
Age, per year	1.03 (1.00- 1.07)	.03	1.01 (0.97-1.05)	.70
Female sex	0.69 (0.32-1.48)	.34	NA	NA
Race (reference, White)				
Black	1.16 (0.56-2.41)	.69	1.08 (0.50-2.35)	.84
Other^c^	2.69 (1.03-7.01)	.04	2.01 (0.74-5.44)	.17
Caregiver (reference, none) (n = 293)	2.33 (1.26-4.32)	.007	2.49 (1.30-4.76)	.01
MAGGIC risk score, per 1-unit increase	1.11 (1.05-1.17)	<.001	1.09 (1.01-1.18)	.03
KCCQ-OS, per 1-unit increase	1.00 (0.98-1.01)	.77	NA	NA
Depression (reference, PHQ-8 score <10) (n = 294)^d^	0.78 (0.37-1.63)	.51	NA	NA
**Model with EI levels of <1.5 and ≥1.5^b^**				
EI ≥1.5 (reference, <1.5)	2.21 (1.20-4.08)	.01	1.98 (1.04-3.77)	.04
Age, per year	1.03 (1.00-1.07)	.03	1.01 (0.97-1.05)	.72
Female sex	0.69 (0.32-1.48)	.34	NA	NA
Race (reference, White)				
Black	1.16 (0.56-2.41)	.69	1.09 (0.50-2.37)	.82
Other^c^	2.69 (1.03-7.01)	.04	1.95 (0.72-5.22)	.19
Caregiver (reference, none) (n = 293)	2.33 (1.26-4.32)	.007	2.47 (1.29-4.73)	.006
MAGGIC risk score, per 1-unit increase	1.11 (1.05-1.17)	<.001	1.09 (1.01-1.18)	.02
KCCQ-OS, per 1-unit increase	1.00 (0.98-1.01)	.77	NA	NA
Depression (reference, PHQ-8 score <10) (n = 294)^d^	0.78 (0.37-1.63)	.51	NA	NA

Abbreviations: AHR, adjusted hazard ratio; HR, hazard ratio; KCCQ-OS, Kansas City Cardiomyopathy Questionnaire overall score; MAGGIC, Meta-Analysis Global Group in Chronic; NA, not applicable; PHQ-8, 8-item Patient Health Questionnaire.

^a^ Explanatory variables with a prespecified *P* value less than .20 in univariable analyses were included in the multivariable analysis.

^b^ EI less than 0.5 indicates discordantly pessimistic; 0.5 to less than 1.5, concordant; and 1.5 or higher, discordantly optimistic.

^c^ Included American Indian or Alaska Native, Asian, multiracial, other or none of the available National Institutes of Health classification options, and unknown or undisclosed.

^d^ PHQ-8 score ranges from 0 to 24, with higher scores indicating more severe symptoms.

**Figure 2.**
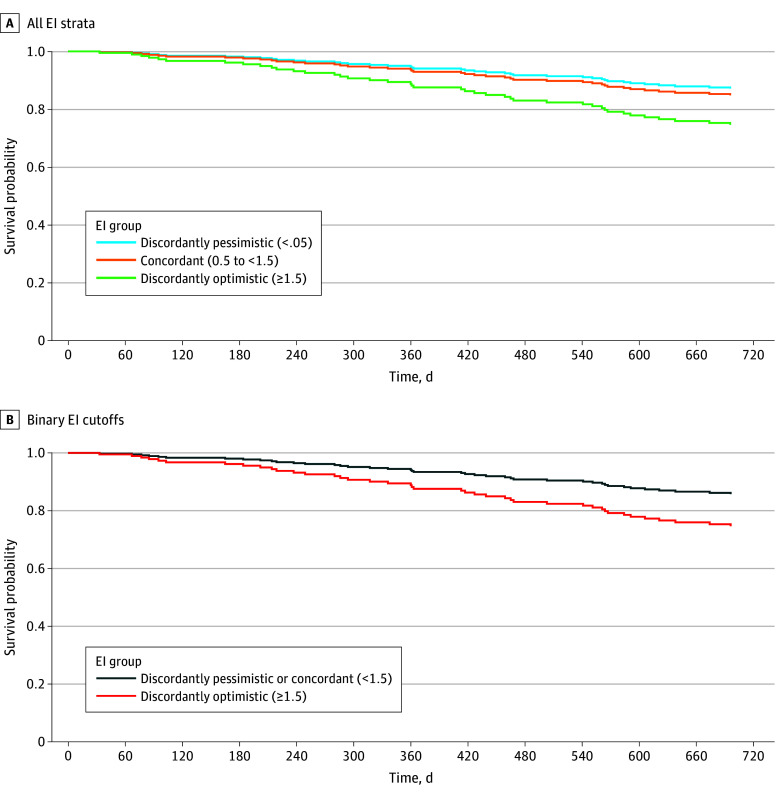
Survival Plot Showing Adjusted Survival Probability by Estimation Index (EI) Survival estimates were adjusted for age, race, caregiver, and Meta-Analysis Global Group Chronic risk score, with receipt of a heart transplant or durable left ventricular assist device as a competing risk.

In the post hoc exploratory analysis using binary EI cutoffs, compared with patients with a discordantly pessimistic or concordant EI (<1.5), those with a discordantly optimistic EI (≥1.5) were more likely to die in the next 2 years (AHR, 1.98; 95% CI, 1.04-3.77), a statistically significant trend (Table 3). The adjusted survival probability by EI strata is shown in Figure 2. Results of the sensitivity analysis examining the association between EI and mortality with KCCQ-OS as a categorical variable were similar to the primary analysis and are provided in eTable 3 in Supplement 1.

## Discussion

In this prospective study of ambulatory, high-risk patients with HF, we identified patient characteristics associated with discordance between self-estimated and SHFM-estimated survival and described the association of mortality with discordance between patient-estimated and model-estimated life expectancy. First, we noted that one-third of patients estimated a life expectancy over 50% longer than the model estimate. Second, factors associated with discordantly optimistic patient-estimated survival compared with model estimates were Black race compared with White race, high MAGGIC score risk, better HF-specific HRQOL, and absence of depression. Third, there was a trend toward increased mortality, progressing from discordantly pessimistic to concordant to discordantly optimistic patient groups, which was statistically significant when the discordantly optimistic group was compared with the discordantly pessimistic and concordant groups. Together, these findings suggest an opportunity to improve patients’ understanding of prognosis, which could enhance shared decision-making for HF therapies.

Previous work has demonstrated discordance between physician- and patient-estimated survival in HF.^25,26,27,28,29^ Ambulatory patients with HF overestimated prognosis, with younger patients and those with increased New York Heart Association class, lower left ventricular ejection fraction, and absence of depression being more likely to overestimate survival.^12^ Our findings add to this literature by identifying patient factors associated with a worse understanding of disease prognosis, including disease-specific HRQOL.

We also identified an association between overoptimism and mortality, but context is needed around how overoptimism was defined. We do not intend to suggest that optimism should be discouraged. Robust literature indicates that optimism, the general expectation that good things will happen, is correlated with improved outcomes.^30,31,32^ Optimism as defined in this study did not explore this concept but focused on the patient’s understanding of prognosis that could lead to a difference in considering advanced therapies. Similar observations have been made in noncardiac diseases such as cancer, where patient overestimation of survival was correlated with differences in treatment selection.^33^ In this context, patient perception of an overoptimistic prognosis may represent a modifiable barrier that could improve informed decision-making for HF therapies.

Of interest, we observed that patients who were most likely to overestimate survival had the shortest average SHFM-estimated survival, and there was an association between an overly optimistic estimate of survival and increasing MAGGIC score (suggesting worse prognosis) and increasing KCCQ-OS (suggesting better self-perceived health status). Furthermore, worse KCCQ-OS was associated with receiving a dLVAD or transplant. While KCCQ-OS is a known predictor of HF survival,^34^ these findings suggest that patients who report worse subjective symptoms despite similar objective HF risk are more likely to receive advanced therapies, supporting the need to holistically and objectively evaluate HF risk and not overly rely on patient-reported symptoms. Sole reliance on patient-reported symptoms could lead to missed opportunities if patients minimize symptoms or could result in overly aggressive treatment among patients who discordantly report worse symptoms despite lower objective mortality risks. Given the overall undertreatment of advanced HF,^35^ the latter may be preferred but could lead to disparities in access to limited therapies, such as transplant.

In addition, patient-estimated survival alone was not associated with survival without considering discordance from the model-estimated survival, which is not surprising given well-described inaccuracies in patients’ understanding of prognosis.^25,26,27,28,29^ The imprecision introduced by incorporating this variable with low prognostic value into a ratio that includes a well-established predictor (SHFM) is a critical feature, rather than a flaw, of this analysis. The goal was not to improve model prediction but to explore the clinical implications of discordance in patient understanding of prognosis. The EI is meant to encapsulate potentially meaningful cognitive or communication gaps that result in discordant patient understanding of prognosis, which is essential to informed decision-making and could be a potentially modifiable measure of alignment of patient perception and clinical reality.

There are several factors, both patient and clinician-related, that likely contribute to poor patient understanding of prognosis. HF has a distinct illness trajectory characterized by exacerbations with recurrent hospitalizations and partial recovery with the possibility of sudden cardiac death at any time.^11^ This contrasts with other illnesses, such as dementia, where there is a continuous decline in health status. While several validated HF survival models exist, such as the SHFM, they tend to overestimate survival.^18,36,37^ There has been significant work dedicated to improving the performance of risk prediction modeling for HF with little gain in accuracy, but there has been little work targeted at helping both patients and clinicians understand the inherent uncertainty associated with HF. Existing literature suggests that clinician estimates of survival with HF are widely variable for the same patient and that physician misunderstanding of prognosis contributes to patient misunderstanding.^15,38^ Furthermore, clinicians feel ill-equipped to discuss prognosis and often lack time to do so, despite the desire of patients with HF and their caregivers for prognostic communications early and repeatedly during the course of the disease.^15^

While several tools are available to facilitate shared decision-making for specific HF therapies such as LVAD,^39^ these tools assume an accurate understanding of the mortality risk from HF. The potential to improve understanding through sharing individualized prognostic predictions with patients is supported by prior qualitative work that found patients valued receiving prognostic information, as it provided clarity, control, and hope.^40^ However, patient-centered tools that facilitate communication about HF-associated illness trajectory and the associated uncertainty to help promote patient preparedness are lacking. Such tools have been used in other chronic illnesses such as cancer, resulting in the improved provision of patient-centered care.^41,42^ Multiple aspects need consideration in developing prognosis communication tools for HF. At the foundation, there is a need to use systematic, best methodologic practices,^43^ with thought to any potential impact on disparities among multiple populations (eg, low health literacy) throughout development and implementation. Caregivers should also be included, as patient cognitive ability declines as HF progresses, making it difficult for patients to make complex decisions.^44^

### Limitations

Our study should be evaluated in the light of several limitations. First, we relied on estimates from the SHFM to assess model-estimated life expectancy, which also overestimates patient survival.^45^ However, to our knowledge this still remains the best available estimate and suggests that the discordance between a discrepantly optimistic patient estimate and actual life expectancy was even larger. Second, we included patients who consented to participate in a prospective registry, were all enrolled at centers with dedicated HF programs, and were willing to share their estimate of life expectancy. Third, univariate screening of potential covariates can bias results toward including covariates whose effect sizes are overestimated, thereby biasing the associations.

## Conclusions

In a prospective cohort study of high-risk, ambulatory patients with HF, one-third of patients overestimated their life expectancy compared with model estimates by more than 50%. Overoptimistic patient estimates of life expectancy compared with model estimates were associated with an increased risk of mortality over 2 years of follow-up compared with patients with more pessimistic life expectancy estimates. These findings suggest that clinicians should holistically and objectively evaluate HF risk when considering advanced therapies, rather than relying solely on patient-reported symptoms.

## Notes

Supplement 1. **eAppendix.** Full REVIVAL Inclusion and Exclusion Criteria **eTable 1.** Univariable and Multivariable Associations for Patient Characteristics and Increasing Optimism Index as a Continuous Variable **eTable 2.** Univariable and Multivariable Associations of Estimation Index and LVAD or Transplant **eTable 3.** Univariable and Multivariable Associations of Estimation Index and Mortality **eFigure.** Correlation Between Patient-Predicted and Model-Predicted Life Expectancy

Supplement 2.
Data Sharing Statement
