# Supplementary material for: Understanding of Prognosis and Estimation of Mortality in Ambulatory Patients With Heart Failure
Source: JAMA Netw Open. 2026 Mar 3;9(3):e260328. doi: 10.1001/jamanetworkopen.2026.0328 (PMC12958085; doi:10.1001/jamanetworkopen.2026.0328)
Supplement: Supplement 1. — eAppendix. Full REVIVAL Inclusion and Exclusion Criteria eTable 1. Univariable and Multivariable Associations for Patient Characteristics and Increasing Optimism Index as a Continuous Variable eTable 2. Univariable and Multivariable Associations of Estimation Index and LVAD or Transplant eTable 3. Univariable and Multivariable Associations of Estimation Index and Mortality eFigure. Correlation Between Patient-Predicted and Model-Predicted Life Expectancy [file jamanetwopen-e260328-s001.pdf]

Supplementary Online Content

Cascino TM, Herron G, Richards B, et al. Understanding of prognosis and estimation of mortality in ambulatory patients with heart failure. *JAMA Netw Open*. 2026;9(3):e260328. doi:10.1001/jamanetworkopen.2026.0328

eAppendix. Full REVIVAL Inclusion and Exclusion Criteria
eTable 1. Univariable and Multivariable Associations for Patient Characteristics and Increasing Optimism Index as a Continuous Variable
eTable 2. Univariable and Multivariable Associations of Estimation Index and LVAD or Transplant
eTable 3. Univariable and Multivariable Associations of Estimation Index and Mortality
eFigure. Correlation Between Patient-Predicted and Model-Predicted Life Expectancy

This supplementary material has been provided by the authors to give readers additional information about their work.

© 2026 Cascino TM et al. *JAMA Network Open*.

eAppendix. Full REVIVAL inclusion and exclusion criteria

Inclusion Criteria

1. Ambulatory.
2. Chronic systolic heart failure ≥ 12 months.
3. NYHA II - IV for at least 45 of the last 60 days.
4. Last documented left ventricular ejection fraction ≤ 35% by any imaging modality.
5. Age 18 - 80 years.
6. Currently under the care of a cardiologist at study site.
7. On appropriate evidenced -based heart failure medications – ACE inhibitor, ARB or sacubitril-valsartan; beta blocker; aldosterone antagonist; hydralazine/long-acting nitrate [required of African-American subjects only] for ≥ 3 months absent contraindications or intolerances.
8. Has ICD or CRT-D. If CRT-D, present for ≥ 3 months.
9. Demonstrated advanced heart failure, including any of the following*:
    i. Serum sodium ≤ 135 mEq/L (obtained as an outpatient)**
    ii. Serum BNP ≥ 750 pg/mL or NT-proBNP ≥ 3000 pg/mL** (obtained as an outpatient)
    iii. Seattle Heart Failure Model (SHFM) one year predicted survival ≤ 85%**
    iv. Heart Failure Survival Score (HFSS) ≤ 7.19**
    v. Peak VO_2 ≤ 55% of predicted for age by Wasserman equation or ≤ 14 ml/kg/min, with RER ≥ 1.05 ***
    vi. VE/VC02 slope ≥ 40***

© 2026 Cascino TM et al. *JAMA Network Open*.

vii. 6 minute walk test (6MWT) distance ≤ 350 m without significant non-cardiac limitation**

viii. Currently listed as UNOS Heart Transplant Status 2 due to heart failure limitation

ix. History of one (1) hospitalization (≥ 24 hours) for acute or acute on chronic heart failure in the past year with either serum BNP ≥ 500 pg/mL or NT-proBNP ≥ 2000 pg/mL** (obtained as an outpatient)

x. History of two (2) hospitalizations (≥ 24 hours) for acute or acute on chronic heart failure in the past year.

10. Willingness to continue to receive heart failure care from the enrolling advanced heart failure clinic over the next two (2) years and to come for all scheduled study visits.
11. Written Informed consent given.

* Qualifying measure must be the most recent of that type of measure obtained (i.e., a BNP ≥ 1000 obtained 2 months prior would not qualify the heart failure subject if a more recent BNP was < 1000)

**Using values obtained within the prior 90 days, except for peak VO_2 within 365 days

***Obtained within the prior 365 days

Exclusion Criteria

1. Known serious medical problem other than heart failure that would be expected to limit 2-year survival (≥50% mortality within 2 years from non-heart failure diagnosis).

© 2026 Cascino TM et al. *JAMA Network Open*.

2. Patient is not likely to be compliant with the protocol, in the opinion of the Investigator.
3. Currently hospitalized.
4. Current use of an intravenous inotrope.
5. Primary functional limitation from non-cardiac diagnosis even if not likely to limit survival.
6. Chronic hemodialysis or peritoneal dialysis or serum creatinine value of ≥ 3 mg/dL at time of enrollment.
7. Cardiac amyloidosis, cardiac sarcoidosis, constrictive pericardial disease, active myocarditis or congenital heart disease with significant structural abnormality.
8. Hypertrophic cardiomyopathy unless dilated LV and no outflow gradient.
9. Cardiac conditions that are amenable to surgical or percutaneous procedures (other than VAD or transplant) that would substantially improve prognosis and for which this subject is a reasonable candidate, regardless of whether the procedure will or will not be performed.
10. Uncorrected hyperthyroidism or hypothyroidism.
11. Pregnancy.

© 2026 Cascino TM et al. *JAMA Network Open*.

eTable 1. Univariable and multivariable associations for patient characteristics and increasing optimism index as a continuous variable.

Variables	Univariable		Multivariable	
	**Back Transformed Estimate[a] (95% CI)**	**P-value**	**Back Transformed Estimate[a] (95% CI)**	**P-value**
**Age per year**	1.01 (0.99-1.01)	0.70		
**Female (reference male)**	0.83 (0.64-1.08)	0.16	1.00 (0.76-1.31)	0.99
**Race (reference White)**				
Black	1.25 (0.94-1.65)	0.12		
Other	0.91 (0.55-1.51)	0.16		
**Caregiver (reference none)**	0.98 (0.76-1.26)	0.98		
**Income (reference** <40k)				
>40k to <80k	0.86 (0.62-1.19)	036		
>80k	1.32 (0.92-1.89)	0.13		
Did not report	1.01 (0.77-1.34)	0.92		
**Education (reference grade school or high school)**				
College or tech school	1.17 (0.86-1.60)	0.32		
Associate /Bachelor's degree	1.23 (0.88-1.71)	0.22		
Graduate degree	1.52 (0.98-2.23)	0.06		
**MAGGIC risk score, per 1 unit increase**	1.01 (1.01-1.02)	<0.001	1.05 (1.03-1.07)	<0.001
**KCCQ-OS, per 1 unit increase**	1.01 (1.01-1.02)	<0.001	1.01 (1.00-1.02)	0.01
**Depression (reference PHQ score <10)**	0.51 (0.40-0.65)	<0.001	0.70 (0.50-0.97)	0.03

Legend: [a]Back transformation represents estimated times change per one unit increase (for continuous variables), or compared to reference group (for categorical variables). For example, for a one-unit increase in the MAGGIC risk score, there is an estimated 1.05 times (or 5.0%) increase in the optimization index. Or for PHQ ≥10, there is an estimated 0.70 times change (or a 30.0% decrease) in the optimization index. Explanatory variables with a prespecified P-value less than 0.20 in univariable analyses were included in the multivariable analysis. Abbreviations: CI – confidence interval; KCCQ, Kansas City Cardiomyopathy Questionnaire; MAGGIC, Meta-Analysis Global Group in Chronic; OR – odds ratio; PHQ, Patient Health Questionnaire

© 2026 Cascino TM et al. *JAMA Network Open*.

eTable 2. Univariable and multivariable associations of estimation index and LVAD or transplant

	Univariable (n=296)		Multivariable* (n=296)	
**Variable**	**HR (95% CI)**	**p-value**	**HR (95% CI)**	**p-value**
**Estimation index (reference low)**		0.79		0.74
Realistic	0.84 (0.45, 1.57)	0.59	1.00 (0.52, 1.90)	0.99
Overestimated	1.02 (0.55, 1.92)	0.94	1.24 (0.64, 2.41)	0.52
**Age, per year**	0.99 (0.97, 1.01)	0.39		
**Female (Reference male)**	0.88 (0.48, 1.60)	0.68		
**Race (ref: White)**		0.20		
Black	0.51 (0.24, 1.07)	0.07		
Other	0.84 (0.26, 2.70)	0.77		
**Caregiver (reference: none), n=293**	1.21 (0.67, 2.22)	0.53		
**MAGGIC risk score, per 1 unit increase**	1.04 (0.99, 1.09)	0.08	1.04 (0.99, 1.09)	0.12
**KCCQ-OS, per 1 unit increase**	0.98 (0.97, 0.99)	0.001	0.98 (0.97, 0.99)	0.001
**Depression (reference PHQ score <10), n=294**	1.22 (0.71, 2.10)	0.48		

*Variables with a p<0.20 in univariable analyses were included in the multivariable analyses

Abbreviations: HR, hazard ratio; CI – confidence interval; MAGGIC, Meta-Analysis Global Group in Chronic; KCCQ, Kansas City Cardiomyopathy Questionnaire; PHQ, Patient Health Questionnaire

© 2026 Cascino TM et al. *JAMA Network Open*.

eTable 3. Univariable and multivariable associations of estimation index and mortality

Variables	Univariable (n=296)		Multivariable (n=293)	
**Model with 3 EI <0.5, 0.5-< 1.5, & ≥ 1.5**	**HR (95% CI)**	**P-value**	**HR (95% CI)**	**P-value**
**Estimation index (reference discordant pessimistic)**		0.04		0.10
Concordant	1.26 (0.52-3.05)	0.60	1.21 (0.49-2.99)	0.68
Discordant optimistic	2.54 (1.12-5.77)	0.03	2.23 (0.94-5.33)	0.07
**Age per year**	1.03 (1.00- 1.07)	0.03	1.01 (0.97-1.05)	0.70
**Female (reference male)**	0.69 (0.32-1.48)	0.34		
**Race (reference White)**		0.13		0.39
Black	1.16 (0.56-2.41)	0.69	1.08 (0.50-2.35)	0.84
Other	2.69 (1.03-7.01)	0.04	2.01 (0.74-5.44)	0.17
**Caregiver (reference none), n=293**	2.33 (1.26-4.32)	0.007	2.49 (1.30-4.76)	0.01
**MAGGIC risk score, per 1 unit increase**	1.11 (1.05-1.17)	<0.001	1.09 (1.01-1.18)	0.03
**KCCQ-OS (reference: 75-100)**		0.92		
**0-49**	1.10 (0.49-2.48)	0.81		
**50-74**	1.16 (0.57- 2.35)	0.69		
**Depression (reference PHQ score <10), n=294**	0.78 (0.37-1.63)	0.51		
**Model with EI <1.5 & ≥1.5**				
**Estimation index discordant optimistic (reference EI <1.5)**	2.21 (1.20-4.08)	0.011	1.98 (1.04-3.77)	0.04
**Age per year**	1.03 (1.00-1.07)	0.03	1.01 (0.97-1.05)	0.72
**Female (reference male)**	0.69 (0.32-1.48)	0.34		
**Race (reference White)**		0.13		0.41
Black	1.16 (0.56-2.41)		1.09 (0.50-2.37)	0.82
Other	2.69 (1.03-7.01)		1.95 (0.72-5.22)	0.19
**Caregiver (reference none), n=293**	2.33 (1.26-4.32)	0.007	2.47 (1.29-4.73)	0.006
**MAGGIC risk score, per 1 unit increase**	1.11 (1.05-1.17)	<0.001	1.09 (1.01-1.18)	0.02
**KCCQ-OS (reference: 75-100)**		0.92		
**0-49**	1.10 (0.49-2.48)	0.81		
**50-74**	1.16 (0.57- 2.35)	0.69		
**Depression (reference PHQ score <10), n=294**	0.78 (0.37-1.63)	0.51		

Legend: Explanatory variables with a prespecified P-value less than 0.20 in univariable analyses were included in the multivariable analysis.
Abbreviations: HR, hazard ratio; CI – confidence interval; KCCQ, Kansas City Cardiomyopathy Questionnaire; MAGGIC, Meta-Analysis Global Group in Chronic; PHQ, Patient Health Questionnaire

© 2026 Cascino TM et al. *JAMA Network Open*.

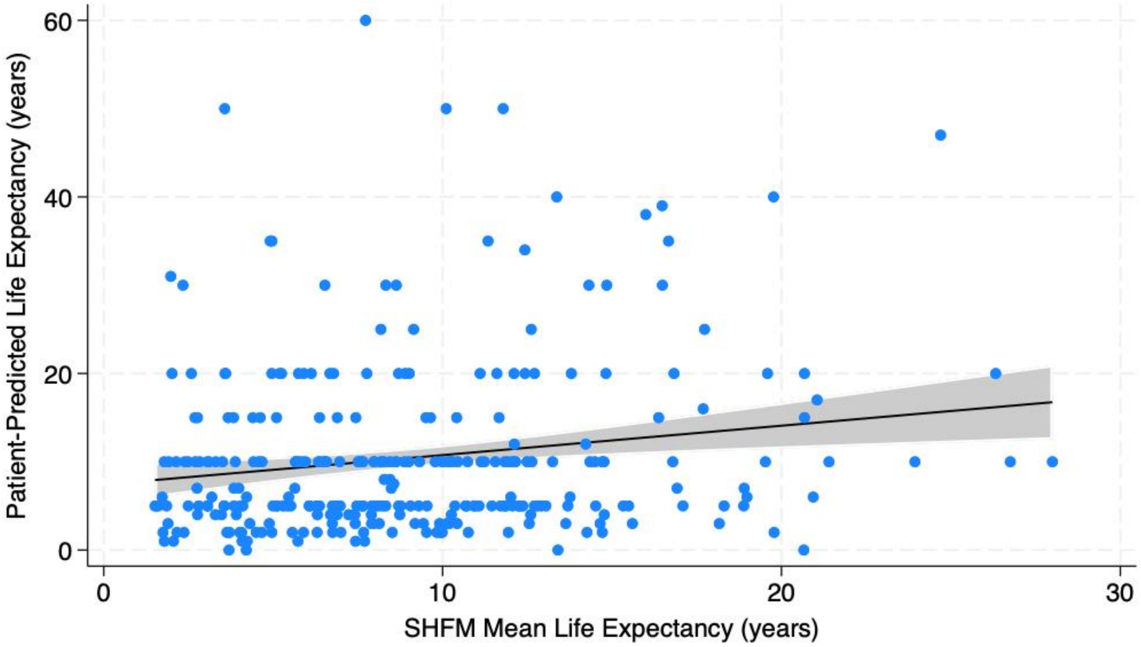

eFigure. Correlation between patient-predicted and model-predicted life expectancy.

Legend: The association between patient-predicted and model-predicted life expectancy is shown in the figure. The correlation between patient-predicted and SHFM model-predicted mean life expectancy was weak (R=0.18, R^2 =0.03, p=0.002).

© 2026 Cascino TM et al. *JAMA Network Open*.
