# Supplementary material for: Understanding of Prognosis and Estimation of Mortality in Ambulatory Patients With Heart Failure
Source: JAMA Netw Open. 2026 Mar 3;9(3):e260328. doi: 10.1001/jamanetworkopen.2026.0328 (PMC12958085; doi:10.1001/jamanetworkopen.2026.0328)
Supplement: Supplement 2. — Data Sharing Statement [file jamanetwopen-e260328-s002.pdf]

Data Sharing Statement

Cascino. Understanding of Prognosis and Estimation of Mortality in Ambulatory Patients With Heart Failure. *JAMA Netw Open*. Published March 03, 2026.
doi:10.1001/jamanetworkopen.2026.0328

Data
Data available: No

Additional Information
Explanation for why data not available: Data available through NHLBI request
